# 

**Published:** 1915-05-15

**Authors:** 


### Personalia.

The Council of the University of Sheffield at its last meeting appointed Mr. J. Sholto C. Douglas, M.A., D.M., B.Ch. (Oxon.), M.Sc. (Birmingham), to the Joseph Hunter Chair of Pathology, in succession to Professor Dean. Dr. Douglas is at present lecturer on pathology in the University of Manchester.

RATES OF SUBSCRIPTION: Twelve months, 6/6; Six months, 4/-; Three months, 2/-, post free to any address in the United Kingdom. Abroad, Twelve months, 8/8; Six months, 5/-; Three months, 2/6, post free.—Address The Subscription Dept., "THE HOSPITAL," The Hospital Building, 28/29 Southampton Street, Strand, London, W.C.

